# Disturbance at the dinner table: Exploring mothers’ experiences of mealtimes when caring for their son or daughter with anorexia nervosa

**DOI:** 10.1177/1359105320904756

**Published:** 2020-02-07

**Authors:** Hannah J White, Emma Haycraft, Iain Williamson, Caroline Meyer

**Affiliations:** 1 Loughborough University, UK; 2 De Montfort University, UK; 3 University of Warwick, UK; 4 University Hospitals Coventry and Warwickshire NHS Trust, UK

**Keywords:** carer, eating disorders, emotions, meals, self-efficacy

## Abstract

This study examined mothers’ (*n* = 9) mealtime experiences when caring for their son or daughter with anorexia nervosa through semi-structured interviews. Interpretative Phenomenological Analysis identified three themes: (1) managing mealtime combat through accommodation and acceptance; (2) feeling isolated, inauthentic and ill-equipped and (3) a need for understanding and to be understood. The overarching concepts of ‘combat’ and ‘distortion’ also underpin the analysis, uniquely outlining how mothers come to understand this daily situation. Mealtime-related interventions need to be developed which prioritise promoting skills and confidence in managing mealtimes and helping carers to address the emotional challenges of these occasions.

## Introduction

Family members of individuals with anorexia nervosa (AN) provide support, detect and monitor disordered behaviours, and assist with accessing and implementing treatment (National Institute for Health and Care Excellence (NICE), 2017; Treasure and Nazar, 2016). However, caring for, or living with, an individual with an eating disorder (ED) has considerable impact on the family (e.g. Fox et al., 2017). Consequently, research has focused on understanding carers’ experiences (e.g. Cottee-Lane et al., 2004). These studies have predominantly focused on overall caring experiences (e.g. Cottee-Lane et al., 2004; Whitney et al., 2005), or parental experiences during a specific period (e.g. during treatment (Tierney, 2005) or recovery (Sharkey-Orgnero, 1999)). Yet experiences and perceived capabilities or competence may vary when considering the overall parenting/caring role, compared with a specific process within that role, such as managing mealtimes. Importantly, Eisler (2005) outlines that family life is often dominated by AN and the resulting intense interactions related to eating and meals, yet little is known about how parents experience the specific mealtime process.

Given the importance of re-nourishment and weight normalisation for individuals with AN (NICE, 2017), family mealtimes have a significant role in recovery. Indeed, Jaffa et al. (2002) suggest that adolescent AN outcome is worse when families do not eat together following treatment. Furthermore, adult AN patients identify mealtime support as helpful in the recovery process (Macdonald et al., 2014). However, mealtimes can be stressful, emotionally intense, conflictual, and last for hours (Fox et al., 2017; Jaffa et al., 2002; Schmidt and Treasure, 2006). Negative emotions related to mealtimes have been outlined by both individuals with AN (e.g. guilt, anxiety, anger; Long et al., 2012) and parents of a child with AN (e.g. ‘extremely stressful’; Cottee-Lane et al., 2004: 173). In addition, parents describe difficult confrontations related to their child’s refusal to eat (e.g. ‘. . .I’d shout, everybody else would cry . . .’; Cottee-Lane et al., 2004: 173). Importantly, family members’ reactions when responding to ED symptoms might maintain the problem behaviours (e.g. Treasure et al., 2008).

Recent treatment guidelines outline that, for all EDs, the needs of family members should be assessed to identify support requirements (NICE, 2017). In addition, carers of individuals with AN have requested support specifically with mealtimes (Haigh and Treasure, 2003). Yet, little is known about how parents manage mealtimes within the home. As parents play an essential role in managing their child’s condition at home, it is important to understand their experiences (Tierney, 2005), which will subsequently provide greater understanding of their mealtime needs.

In summary, in the context of AN, supportive mealtimes are considered beneficial for recovery (Jaffa et al., 2002; Macdonald et al., 2014). However, mealtimes can be a stressful and challenging time for parents (Cottee-Lane et al., 2004). Although research has explored broader parental experiences of caring for an individual with AN (e.g. Whitney et al., 2005), little has focused specifically on understanding the experiences of mealtimes within the home. The importance of involving all family members (i.e. parents and siblings) in the treatment of AN has been acknowledged (e.g. Herpertz-Dahlmann et al., 2015), however, given that mothers typically take primary responsibility at mealtimes (Sepúlveda et al., 2012), they were the focus of the current study. The main research question was: *How do mothers experience family mealtimes when caring for a young person with AN?* Interpretative Phenomenological Analysis (IPA; Smith and Osborn, 2003) was used as this allows for exploration of what mothers understand about these situations, their interactions and their roles.

## Method

### Participants

Nine mothers (aged 43–53 years; *M* = 48.9, *SD* = 3.8; see Table 1 for full demographics) participated in this study, responding to adverts placed at ED charitable services or on research websites. Participants all identified that their child (daughter, *n* = 7; son, *n* = 2) had either received, or was currently receiving, treatment for AN. Child age ranged from 16 to 24 years (*M* = 19.6, *SD* = 2.7). Mothers reported that help was first sought between the ages of 12 and 21 years (*M* = 15.2, *SD* = 2.9). All children had received treatment (outpatient only, *n* = 3; inpatient and outpatient, *n* = 6). Three mothers felt their child had ‘recovered’ from their AN, the majority of mothers felt that their child was either still unwell or recovering (*n* = 6). Where mothers reported that their child had recovered, they discussed family mealtimes when their child was unwell. The majority of mothers were married or living with a partner (*n* = 7) and had other children (*n* = 7).

**Table 1.** Participant demographic information.

Pseudonym	Age	Marital status	Sex of child	Age of child (years)	Child’s age (years) when first seeking professional help	Type(s) of treatment child has received
Agnes	53	Married	Female	17	12	Inpatient and outpatient
Betty	47	Living with a partner	Male	20	17	Outpatient
Cathy	51	Married	Female	17	13	Outpatient
Dawn	51	Married	Female	24	21	Inpatient and outpatient
Esther	43	Divorced	Female	16	12	Inpatient and outpatient
Frances	43	Single	Male	19	14	Inpatient and outpatient
Gina	52	Married	Female	23	16	Inpatient and outpatient
Hollie	51	Married	Female	20	15	Inpatient and outpatient
Isla	49	Married	Female	20	17	Outpatient

### Procedure

Following ethical approval and informed consent, semi-structured interviews (telephone or face-to-face) were conducted individually by one of two researchers. The interview schedule included topics such as mothers’ mealtime experiences and feelings related to these, management of mealtimes at home (considering food consumption, and the young person’s anxiety levels and behaviour), involvement of the young person and other family members in the mealtime, and advice and support about mealtimes (full schedule available on request from the corresponding author). Interviews lasted between 40 and 93 minutes (*M* = 69 minutes, *SD* = 20.5) and were transcribed verbatim.

### Data analysis

Data were analysed using IPA (Smith and Osborn, 2003). IPA aims to explore how participants make sense of particular personal experiences and, specifically, their accounts and perceptions of events (Smith and Osborn, 2003). The active role of the researcher within the analysis process is also recognised. For example, themes generated from the data occur through researchers’ interpretations as they make sense of the participant’s experience (Smith, 2004). Importantly, IPA utilises a small and purposive sample (Smith et al., 2009); this enables the recruitment of a fairly homogeneous sample, where individuals all share key characteristics which are relevant to the research aims. It should be noted that theme saturation is not the focus of IPA, instead the in-depth engagement with a small number of cases allows for full and detailed interpretation of the data and the examination of divergence and convergence (Brocki and Wearden, 2006; Smith and Osborn, 2003). IPA has previously been used to examine the experiences of mothers within the context of adolescent AN (Bezance and Holliday, 2014).

The analysis followed the guidelines described by Smith and Osborn (2003). An idiographic approach was utilised, conducting the analysis initially on a case-by-case basis before conducting a cross case analysis (Smith, 2004). The analysis process initially included familiarisation, early annotations and recording emergent themes. Next, themes were connected, creating a list of themes for the first case. This process was repeated with the other cases (each case being analysed independently) and a list of themes for each participant was created. Shared themes were then identified before final master themes were constructed and specific extracts selected from the transcripts (Smith and Osborn, 2003). In selecting extracts, the analysts aimed to illustrate central conceptual elements of the experience under scrutiny while recognising and reporting subtle differences within the participants and making sure that all voices were represented in a broadly equitable manner. Both elements are key to ensuring the analysis is true to the idiographic sensibility of the analysis (see Smith et al., 2009).

Credibility checks of the analysis and findings were conducted (Elliott et al., 1999). Specifically, four transcripts were reviewed and the analysis was overseen by a second qualitative researcher who acted as an ‘auditor’ (Elliott et al., 1999: 222). There were no disagreements on final themes.

## Results

Mothers’ mealtime experiences and associated emotions and behaviours are presented across three themes. The first theme ‘*Managing mealtime combat through accommodation and acceptance*’ largely covers some of the behavioural challenges described by mothers around mealtimes. The second theme ‘*Feeling isolated, inauthentic and ill-equipped*’ describes the emotional consequences mothers experience during mealtimes with their child. Finally, the third theme, ‘*A need for understanding and to be understood*’ explores some of the strategies mothers report using to try to manage their situation.

These three themes are enriched through two super-ordinate recurrent thematic concepts; ‘combat’ and ‘distortion’ that represent core quotidian components of the participants’ experience. The first is presented through inter-related themes which consider the nature of that combat from the mothers’ perspectives and the consequences for their self-image and relationship with their children. ‘Distortion’ also underpins the analysis, where mothers describe adapting behaviour and cognitions to their new artificial reality. In all cases our interpretations are illustrated with a series of quotations from the transcribed interviews. All names used are pseudonyms.

### Managing mealtime combat through accommodation and acceptance

Mealtimes, in the context of their child’s AN, were invariably reported as extremely difficult experiences, described by various participants as being ‘*absolutely horrible*’, ‘*really stressful*’, ‘*painful*’ and ‘*frightening*’. Metaphors of mealtimes as a ‘*battlefield*’ and the use of ‘battle’ language permeated mothers’ accounts and acknowledges the challenges and challenging behaviour mothers often face throughout the mealtime process. These challenges were present within pre-meal activities (e.g. planning, shopping and preparation), during the eating phase of the meal, and for some, supervising their child after the meal to prevent vomiting.

Unsurprisingly, mothers reported that their child’s illness and the related ED behaviours were the source of much conflict at mealtimes. Reported behaviours included, food refusal, hiding or throwing of food, spitting food out into napkins, excessive label checking, controlling behaviour in relation to other family member’s food, drinking large quantities of water, deception and aggression. Mothers frequently reported feelings of ‘*anger*’ and ‘*frustration*’. They also reported additional challenges when their child developed new ED behaviours: ‘*. . .it was like oh something new . . . they’ve learnt to do this today . . . something else chucked into the mix, oh for god’s sake . . .*’ (Betty).

In relation to managing some of the mealtime challenges, mothers described how they would try to avoid mealtime conflict. This was often in relation to prioritising getting some food eaten over challenging specific disordered behaviours. With this focus on the food, there was a sense of ‘avoiding the fight’ and ED behaviours were accommodated. This involved playing along with a distortion of reality with mothers giving the pretence that behaviours which signified a serious eating issue were actually routine and mundane

. . .I don’t take any notice because I think as far as I’m concerned as long as she’s eating and keeping it down I don’t really care how she gets there. (Gina)

Oh it’s horrible, I hate it, it’s absolutely unreal, it’s just an atmosphere in my head that you know I can’t bear the food, I can’t bear the way it’s dealt with, you know the portion sizes are measured out and every grain is counted and oh everything but if it means that she will eat the meal then that’s what we all toe the line and do. (Hollie)

Hollie’s upset in this situation and her dislike in colluding in a distorted ‘reality’ are clearly apparent. However, she suggests that if the meal does not happen in this way, her daughter may not consume the food and, as a result, any challenges to this process are supressed and the disordered behaviours are accepted for ‘the greater good’. Hence, there is an aspect of pretence that this situation is acceptable and normal despite Hollie acknowledging that it is ‘*absolutely unreal*’ and internally managing her feelings of frustration. In addition, ‘*we all toe the line*’ signifies that it is the *whole* family who obey these ‘rules’ to get the food eaten by her daughter, highlighting the level of control the illness is seen to possess within the family, especially around mealtimes.

Conflict was also avoided due to feeling as though the battle against AN had already been lost. Frances explains that, guided by health professions, she was told not to challenge her son’s behaviour to avoid mealtime conflict; she should instead pretend and promote the illusion that his behaviour is acceptable

. . .the paediatrician said to me ‘. . .do not force him, don’t make mealtimes you know a warzone or a battleground, . . . make them as calm and as stress-free as possible so that he gets the impression that you think that what he’s doing is fine . . .’

Associated with this advice, Frances also described how she accepted and accommodated to the situation

. . .just when you think they’ve done something bizarre and you think ‘oh my god’ you know they’ll go and do something else and you just have to accept it really, you know, as far as mealtimes go . . . I think that’s all there is . . . there’s no limit to how bizarre things can get . . .

Frances’ account of acceptance suggests that this is the new normal; there is no scope to challenge the status quo and no battle left to fight – even when pushed to the boundaries of disbelief and despair. Similarly, Agnes highlights feeling defeated and unable to challenge in the battle to get her daughter to eat. This highlights the strength of the AN and again suggests learning to tolerate the situation, and that the battle is lost

. . .she used to block you and just say ‘no’ and you knew that there was literally no point in having the battle because you weren’t going to budge her, you weren’t going to win, and she got to the point where she would get hysterical and quite violent. . .

### Feeling isolated, inauthentic and ill-equipped

*Being a parent* and *being a carer* were recognised as separate, potentially conflicting, roles. Agnes acknowledges that ‘*to try and be parent and carer, both at the same time, I think is enormously difficult*’. For example, one difficulty outlined by Isla was the loss of genuine ‘mum time’ as all shared time focused around encouraging eating – which her daughter resented. This outlines the complexity of how mothers may see themselves, and their role – forcing her daughter to do something she hates may clash with her self-image of a caring mother

. . .she’d say ‘all the time we get together mum, it’s only, I’m only ever eating and I hate eating, we never do anything apart from eating’. . . so I guess that’s why I was enemy number one.

Within this theme, participants described how they felt about managing the joint roles of parent and carer, and some of the challenges that arose. Some mothers had developed ideals regarding how they felt they should behave and feel during mealtimes. In trying to achieve these behavioural expectations, mothers reported creating an unnatural reality which led to feeling inauthentic and unspontaneous as a mother:

I did feel that I . . . kind of became to be an actor, that it was very hard and it didn’t come naturally to me . . . You can’t just switch off, you have to think ‘right what am I supposed to be doing now?,’ ‘what am I supposed to be saying about this?’. You know you have to try and remember what you’ve read or what you know, so it’s all a bit artificial really. (Dawn)

The reference to acting highlights the contrived nature of the situation, and builds on the initial discussion of pretence in theme one. Dawn describes trying, and struggling, to enact the ‘correct’ behaviours for mealtimes. However, despite her attempts to minimise the ‘artificiality’ of the situation (‘a bit’), this continual evaluation of her behaviour may also represent a deeper sense of despair about the situation.

In addition to behavioural expectations, emotional expectations were also reported. Mothers reported how their own feelings could influence their child and the mealtimes. Consequently, mothers reported a need to remain emotionally in control and calm, in line with advice received. In attempts to minimise any impact their own emotions had on the situation, there was often a level of pretence, with emotions (both positive and negative) being consciously hidden. Frances describes her joy at her son eating a different food; however, she reports feeling unable to share this with him

. . .I’d be doing a little jig in the kitchen, you know, because he might have eaten something that he hadn’t eaten before . . . but I kept it completely to myself, I didn’t even let him see that I was pleased because . . . I didn’t want him to think . . . that it was a big deal. . .

Frances’ concern about how her son will interpret her emotions leads her to contain them and present a controlled appearance. However, difficulties were also expressed by mothers regarding hiding emotions and keeping in control, particularly when presenting an expressionless façade was ‘*so contradictory*’ to the fear and worry they felt about the situation.

Several additional challenges were expressed in relation to parents being carers. There was a sense of being unarmed and under-resourced – without strategies or confidence to deal with challenging behaviour. Many mothers expressed a lack of confidence in their caring role, as outlined by Dawn who reports ‘*I’m just a parent, what do I know?*’. Challenges often included feeling ill-equipped within this role due to having *‘totally no experience’.* This often resulted in feelings of uncertainty regarding their caring role and if they were doing it correctly

. . .you don’t know whether you’re doing it right or not, and you know nobody could tell us if what we were doing was right and, you know, is there a right or a wrong? It’s really hard to know so your self-doubt is huge. . . (Cathy)

Cathy depicts a sense of desperation about the situation and her actions. Questioning ‘*is there a right or a wrong’* way of being a carer emphasises her desire for clarity and direction. Yet, with no-one addressing her feelings of uncertainty or normalising the situation, this also seems to be impacted by loneliness.

These feelings of uncertainty necessitated that mothers improvised and created their own strategies to manage the situation. Agnes describes feeling lost and struggling to know what to do, and also wanting to move forward. She also portrays an idea of isolation; that she has to act herself as she is on her own with no one to help

. . .there wasn’t actually anybody helping you with a strategy. It really was floundering around on your own . . . you kind of made up the rules as you went along and did what you thought might get you somewhere. . . (Agnes)

Several interviews included discussions regarding inpatient care and the challenges of encouraging food consumption at home, in their role as the main carer, versus in hospital, by trained professionals. One difficulty identified was awareness that, as a parent, there were limitations regarding providing consequences for disordered behaviour

. . .she generally ate the food that was in front of her and you think well actually as a parent, if I’d have confronted them to that point and said ‘right you eat it’ but what would I have said as a parent about the ‘or else’?. . . (Agnes)

Agnes highlights how by being a parent within a caring role she feels ill-equipped to manage any challenging behaviour; without clear answers she portrays feeling unarmed. This was also reflected by Gina, who described physical limitations and feeling powerless in trying to stop her daughter going to the bathroom to vomit after meals; ‘*I wasn’t going to restrain her or anything like that*’. Challenges were also identified in the transition of care from a hospital environment with a structured feeding plan to the ‘*real world*’ at home

. . .really the hard bit was when they got home trying to maintain you know the good work that they had done in a real-life situation because it was a very artificial situation that they were in in an eating disorders unit . . . you can’t replicate that in the real world so that’s when the hard work started. (Gina)

Gina outlines the difficulties with trying to replicate hospital procedures at home and highlights the differences between the two environments and the subsequent challenges with this transition period. She also suggests that hospital and home are two separate worlds; an artificial situation and real-life. However, as we see within the data, the artificiality of the clinical world bleeds into the way mealtimes are performed in the domestic realm with pretence and denial being employed as coping strategies and even endorsed by health care professionals. Essentially the survival need of the children to eat sufficient food dominates the ability of the mothers to behave in an authentic manner.

### A need for understanding and to be understood

Within this theme mothers identified some of the strategies they found helpful, and also described some aspects which they felt would have been useful in their role as carers. These focused on getting educated and seeking support. Some mothers expressed initially having a lack of understanding of AN. Dawn highlights the complexity of the disorder: ‘*There’s just so many sides to it . . . it’s just a big lot of confusion really*’. Therefore, one strategy carers reported as helpful was to increase their understanding of AN:

. . .it was difficult and as I say I did feel isolated and helpless . . . I just dealt with it the way I knew how and that was to arm myself with information. . . (Frances)

Through another conflict metaphor, Frances highlights how by addressing her feelings of helplessness and in preparation to battle with her son’s anorexia, she arms herself with information. This portrays knowledge and understanding of the illness as a powerful weapon. This is supported by mothers’ descriptions of how an increased understanding of AN influenced their caring role. For example, Dawn explains that ‘*once you start learning about anorexia you realise how they are cheating the system sort of and doing things you didn’t realise*’. Knowledge is again shown to be powerful here as through increased understanding of anorexia, there is also the increased awareness of ED behaviours being conducted which were previously not recognised. This recognises the need to appreciate more fully elements of deceitfulness in their child’s behaviour and to tackle this or – perhaps more typically – to accept this (which is a peculiarly ‘un-motherly’ act).

Similarly, Gina outlines the power of knowledge; being educated about her daughter’s illness helped with providing support to her daughter and also managing her own emotions

. . .being educated by the eating disorders unit helped us an awful lot with that understanding and that really helped us an awful lot with managing the feelings of frustration and understanding how she must feel and supporting her. (Gina)

Mothers also spoke of the positive experiences of talking to other parents in similar situations, they sought solidarity. This was particularly important as medical professionals were outlined as having *‘objective’* rather than personal experiences of EDs

. . .I felt very isolated and I felt this need . . . for it to be normalised because it was our reality, it was our life and I just almost like I needed somebody else to say, to sort of validate it and to say ‘that’s fine, yeah he’s doing that but that’s fine, my daughter or my son does that . . .’ instead of me telling my friends whose children didn’t have anorexia and for them to be sort of raising their eyebrows and saying ‘blimey, oh god, you know that’s a bit weird isn’t it’. . . (Frances)

Frances outlines the profound loneliness of her situation and how she wants reassurance that her son’s behaviours and their *‘life’* are typical within the context of an ED. Evident again here is the concept of adapting mothering within a distorted reality and a need for this ‘new normal’ to be understood and supported. Reinforcement was another area of desired support described by Agnes:

. . .I don’t think you can take away from how difficult it is but I think you can have somebody that is your sort of backstop that keeps putting you back in the frame as it were and dusting you down and patching you up and putting you back on the frontline and that’s what would have been helpful I think

This extract highlights the relentless and difficult nature of mealtimes. Reference to *‘patching you up’* suggests being wounded by the mealtime process and needing assistance to continue the ‘battle’ with AN.

Mothers also described the benefit of solidarity, particularly from other carers understanding how they felt. Betty describes how this empathy helped her mood and also provided validation and humanisation of her experiences

. . .it’s ok to feel like that, you’re not on your own, you’re not the only one and it’s almost like, take a step back, take a breather and you know try not to get wound up by it, and you know, you felt better that you were being . . . told by them that had been there, done it, got the t-shirt . . . you were a real person and John was a real person . . ., you weren’t just a number. (Betty)

## Discussion

This study explored mothers’ experiences of mealtimes when caring for their child with AN. Three themes were identified. The first (*Managing mealtime combat through accommodation and acceptance*) captures the difficult and demanding nature of mealtimes; particularly managing ED behaviours. Accommodating ED behaviours was commonplace and attributed to feeling defeated by the illness or mothers colluding with the child and ‘picking their battles’ to minimise conflict and prioritising some level of eating. The second theme (*Feeling isolated, inauthentic and ill-equipped*) describes low confidence and uncertainty regarding the caring role. These feelings were associated with difficulties adhering to their own expectations of behaviour and emotional restraint during mealtimes, and lacking mealtime support and guidance. Within the third theme (*A need for understanding and to be understood*), mothers described strategies they found beneficial when managing mealtimes. Increasing understanding about AN was viewed positively and helped to promote mothers’ confidence and empowerment. Meanwhile, mothers reported positive experiences of seeking solidarity from other carers to help normalise their experiences. As mentioned above, interwoven through the themes are superordinate concepts of ‘combat’, and uniquely, the aspect of ‘distortion’. The first considers the nature of combat and the ‘battle’ with AN from the mothers’ perspectives, while the latter highlights how mothering is adapted within this new distorted reality, and the need to understand and normalise the situation. These two processes appear to represent the most emotionally upsetting elements of mealtime experiences with women’s self-image as a ‘good’ mother being threatened. Although conflict avoidance strategies serve to reduce expressed emotion at mealtimes, they typically involved mothers moving into an inauthentic model of parenting where they collaborate with an often-personified anorexia by engaging in a pretence that their child’s maladaptive eating patterns are perfectly normal. This involves an avoidance of interpersonal disturbance but replaces it with an intra-psychic sense of disturbance and sense of a mother who is not performing her role at all well. This may be particularly difficult to tolerate when other family members, especially siblings of the child with AN, are involved.

The current findings support and extend previous work highlighting how challenging mealtimes within the context of AN can be (e.g. Cottee-Lane et al., 2004) and that carers often tolerate difficult behaviours and collude with the individual with AN to avoid conflict (Fox and Whittlesea, 2017). As accommodation of behaviours and cognitions can unintentionally maintain the illness (e.g. Treasure and Schmidt, 2013), it is important to understand how and why carers accommodate ED behaviours. Fox and Whittlesea (2017) suggested that it may be due to carer ‘burnout’, to which low self-efficacy may contribute. Indeed, within the current study, mothers’ beliefs in their capabilities (or self-efficacy; Bandura, 1997, 2006) were frequently referred to, for example their competence in managing emotions or successfully challenging ED behaviours. Importantly, the results are novel in identifying some of the reasons *why* carers may have low self-efficacy in addressing anorectic behaviours during mealtimes. For example, mothers used comparisons with healthcare professionals and inpatient care, outlining a lack of consequences for behaviours and the pressure to try to replicate the artificial hospital mealtime environment at home. It is also important to acknowledge that the circumstances are described as being a new and very different reality for these mothers. Even those who have themselves experienced eating difficulties have not previously faced the situation as a parent. Focusing on what is new or different in tasks, instead of what is the familiar, and within one’s capabilities, might undermine self-efficacy and existing skills and knowledge (Bandura, 1982). Hence, this strange, new situation may instill feelings of incompetence and support should be given to parents regarding developing new skills and competencies to manage the additional demands they face.

This study identified factors which mothers felt were beneficial for managing mealtimes. Knowledge was shown to be a powerful tool in relation to developing skills but also helping to manage emotions – a particularly important aspect within the caring role (Treasure et al., 2007). In addition, mothers also highly valued the support from other carers who had been there themselves, and our findings extend past research (Fox et al., 2017), by identifying key aspects of mealtime support, such as wanting their child’s food-related behaviours and their experiences normalised within the context of AN, and their own feelings about the situation validated. Providing parents with such support may increase confidence and efficacy within the caring role.

This can be achieved in a number of ways by considering sources of self-efficacy beliefs (Bandura, 1977). First, carers need practical help and support to achieve a sense of ‘mastery’ over mealtimes, this could be achieved via more information about ED behaviours, promoting understanding of accommodating behaviour, or practical support managing challenging mealtime behaviour. Second, resources would benefit from success stories from other carers who have experiences of mealtimes within AN. Third, healthcare professionals should encourage and promote carers’ skills and confidence in their abilities to manage mealtimes. In particular, the transition from inpatient to home might be an important time for ED units to work with families to provide education and promote self-efficacy in relation to mealtimes. Finally, emotional responses to mealtimes are a prominent feature of a parent caring for a child who has AN and further support is needed to help carers to manage their emotions during this time.

Strengths of this study include the in-depth focus on the experiences of a number of mothers and the identification of numerous important themes relating to their mealtime experiences. It is noted that as IPA focuses on exploring the experiences of a specific group of people, a small and homogeneous sample is required (Smith and Osborn, 2003). This general requirement, along with a consideration that participating mothers responded to recruitment adverts and may have been particularly motivated to take part in the research, may limit the transferability of the findings to other mothers who may experience mealtimes with their child with AN differently, especially those who do not have and may not seek peer support. In addition, as details relating to the young person’s body mass index (BMI) or subtype of AN were not collected, it should be acknowledged that these may influence the dynamics – and challenges – at mealtimes in different ways (e.g. the presence of purging behaviours). To assist with promoting carers’ confidence and efficacy with managing mealtimes, consideration should be given to identifying any specific mealtime challenges carers may face related to their child’s diagnosis of AN.

Mealtimes are clearly a challenging time within the context of AN. The need for practical mealtime support, development of mealtime interventions, and the teaching of behavioural techniques to address nutrition and mealtime challenges has previously been identified (Haigh and Treasure, 2003; Macdonald et al., 2014; Sepúlveda et al., 2008). However, these findings highlight deficiencies in currently available support systems and indicate a priority area to target in relation to improving AN treatment. In addition, the importance of helping carers to hone their emotional intelligence skills has been previously outlined (Treasure et al., 2007), however, the current findings also uniquely highlight that carers need support to manage emotions specifically within the mealtime environment. The development of carer resources should focus on promoting self-efficacy with the mealtime process and how to manage emotions at this time. Future research should explore these concepts among carers of an individual with AN, considering the different subtypes of AN, and particularly in relation to treatment outcomes. There is also the potential to explore accounts of mealtimes in the context of AN from different or even multiple perspectives (the individual with AN, parents and potentially siblings). Rather than using one-off interviews, using an ongoing method of capturing data at regular intervals and closer to real time (such as using various forms of diary keeping – see Gough and Lyons (2016) and Williamson et al. (2015)) as well as, or instead of traditional interviews, would be advantageous for future research to implement.
